# The effects of alcohol consumption, psychological distress and smoking status on emergency department presentations in New South Wales, Australia

**DOI:** 10.1186/1471-2458-7-46

**Published:** 2007-04-02

**Authors:** Devon Indig, Margo Eyeson-Annan, Jan Copeland, Katherine M Conigrave

**Affiliations:** 1 National Drug and Alcohol Research Centre, University of New South Wales, Sydney, Australia; 2 Centre for Epidemiology and Research, NSW Department of Health, 73 Miller Street, North Sydney, Australia; 3 Drug Health Service, Royal Prince Alfred Hospital, Missenden Road, Sydney, Australia

## Abstract

**Background:**

Despite clear links between risky alcohol consumption, mental health problems and smoking with increased morbidity and mortality, there is inconclusive evidence about how these risk factors combine and if they are associated with increased attendance at emergency departments. This paper examines the population-level associations and interactions between alcohol consumption, psychological distress and smoking status with having presented to an emergency department in the last 12 months.

**Methods:**

This study uses data from a representative sample of 34,974 participants aged 16 years and over from the New South Wales Population Health Survey, administered between 2002 and 2004. Statistical analysis included univariate statistics, cross-tabulations, and the estimation of prevalence rate ratios using Cox's proportional hazard regression model.

**Results:**

Results show that high-risk alcohol consumption, high psychological distress and current smoking were all significantly and independently associated with a greater likelihood of presenting to an emergency department in the last year. Presenting to an emergency department was found to be three times more likely for women aged 30 to 59 years with all three risk factors and ten times more likely for women aged 60 years or more who reported high risk alcohol consumption and high psychological distress than women of these age groups without these risk factors. For persons aged 16 to 29 years, having high-risk alcohol consumption and being a current smoker doubles the risk of presenting to an emergency department.

**Conclusion:**

The combination of being a high-risk consumer of alcohol, having high psychological distress, and being a current smoker are associated with increased presentations to emergency departments, independent of age and sex. Further research is needed to enhance recognition of and intervention for these symptoms in an emergency department setting in order to improve patient health and reduce future re-presentations to emergency departments.

## Background

The effects of excessive alcohol consumption, mental health problems, and smoking status on morbidity and mortality are well documented. A number of studies have reported the extent of emergency department presentations related to alcohol consumption, psychiatric conditions, and tobacco consumption [1-6]. However, uncertainty remains about the population-level associations and interactions of these risk factors with emergency department presentations. Some studies have reported an inverse relationship (the more someone drinks, the less likely he or she is to seek healthcare) [7], some have found no association [8], and others have found that problem drinkers access emergency departments more often than non-problem drinkers [9]. Few studies have investigated the population-level association between psychological distress and the use of emergency services. One important measure of psychological distress is the Kessler-10 (K-10) scale, which measures rates of self-reported anxiety and depression in the adult population [10-12].

It is important to gain a better understanding of the independent and interactive effects of these risk factors and how they relate to emergency care in order to develop targeted prevention and intervention strategies. Developing an intervention which targets groups with multiple risk factors can help ensure the maximum benefit from programs with limited resources. The objective of our study was to examine the population-level associations and interactions between alcohol consumption, psychological distress, and smoking status, with presenting to an emergency department.

## Methods

### Study design

This study reports on a secondary analysis of data collected from the New South Wales Population Health Survey, conducted by the Centre for Epidemiology and Research, NSW Department of Health, using Computer Assisted Telephone Interviewing (CATI) [13]. This survey was designed to monitor self-reported health behaviors, health status, health service use, satisfaction with health services, and other factors that influence health. The conduct of the New South Wales Population Health Survey was approved by the Ethics Committee of the NSW Department of Health. The current analysis was conducted to determine the associations and interactions between two health behaviors (alcohol consumption and smoking status), a health status (psychological distress), and a health service use (presentation to an emergency department in the last 12 months).

### Sample

The target survey population included all residents of New South Wales living in households with private telephones between 2002 and 2004. A high percentage (97.5%) of all residences in Australia have landline telephones [14]. Households were sampled using list assisted random digit dialing. One person was randomly selected from each household contacted for interview. Most interviews (99%) were conducted in English but the survey is also conducted in five other languages: Arabic, Chinese, Greek, Italian and Vietnamese. The final sample size included 34,974 participants aged 16 years and over. The overall response rate for the three years was 66%. This response rate is comparable with other population-wide surveys using similar sampling strategies such as the Behavioral Risk Factor Surveillance System in the United States which had a median 52.7% response rate in 2004[15].

### Outcome measures

The main outcome measure of our study was whether or not the survey respondent indicated they had presented to an emergency department in the last 12 months. Respondents were asked: 'How often do you drink alcohol?' and 'On a day when you drink alcohol, how many standard drinks do you usually have?'. With the assistance of a conversion chart, respondents estimated their usual alcohol consumption in standard drinks. The study categorised alcohol consumption using the National Health and Medical Research Council's *Australian Alcohol Guidelines *[16]. Categories were derived based on the reported usual number of standard drinks per occasion, including: non-drinkers, low risk (1–2 standard drinks if female, 1–4 if male), risky (3–4 standard drinks if female, 5–6 if male) and high risk (5 or more standard drinks on any occasion if female, 7 or more if male).

Psychological distress was measured using the Kessler Psychological Distress Scale (K-10) [10] which consists of questions about symptoms of anxiety and depression in the previous four weeks. Levels of psychological distress were established based on scale scores, including low (< 16), moderate (16–21), and high (22 or more) [17]. Respondents who indicated they smoked daily or occasionally were categorized as current smokers. Ex-smokers were identified as respondents who indicated they do not smoke now but used to, or have tried it a few times but never smoked regularly. Non-smokers were people who indicated they have never smoked.

### Weights

Data were weighted in the analysis to adjust for age, sex and geographical differences in the probabilities of selection among participants. These differences were due to variability in a number of areas including: the number of people living in each household, the number of residential telephone connections, and the sampling fraction in each health area. Survey estimates were adjusted using post-stratification weights to reduce the effect of differing non-response rates among males and females and different age groups [18].

### Statistical analysis

Statistical analysis was performed using SAS version 8.02 and SUDAAN 9.0 (software for survey data analysis) [19,20]. Univariate statistics and cross-tabulations were analyzed using the SURVEYMEANS procedure in SAS version 8.02. The SAS-callable version of SUDAAN was used to measure the associations and interactions between the variables of interest. Logistic regression was considered in the analysis since the odds ratios it estimates can be used to approximate relative risk. However, this method was not used since it is only appropriate for a rare event. With the large survey sample size and a common outcome event, Cox's proportional hazard regression model using the SURVIVAL procedure in SUDAAN was used as a more appropriate way to estimate relative risk. By assuming a constant risk period (time fixed at 1), the conditional hazard ratio estimated by Cox's model can be adapted to estimate the prevalence rate ratio [21]. Prevalence rate ratios were calculated for each risk factor (alcohol consumption, psychological distress and smoking status) individually and combined in order to estimate the interaction effects. These results were then stratified by sex and age groups to identify demographic trends.

## Results

### Sample characteristics

Table 1 describes the characteristics of the survey respondents, including the: total sample (N = 34,974), high-risk alcohol consumers (N = 2,187), highly psychological distressed respondents (N = 3,259) and current smokers (N = 7,113). When comparing these populations, males are more likely to be high-risk consumers of alcohol and current smokers than females, while females are more likely to have high psychological distress than males. Respondents under the age of 40 and those who have never married are more likely to have high-risk alcohol consumption, high psychological distress and be current smokers than older or married respondents.

**Table 1:** Socio-demographic characteristics by risk factor in NSW, 2002–2004

	**High risk alcohol consumption^a ^% (N = 2,187)**	**High psychological distress^b ^% (N = 3,259)**	**Current smoking^c ^% (N = 7,113)**	**Total population % (N = 34,974)**
**Sex**				
Male	58.1	42.2	54.3	49.2
Female	41.9	57.8	45.7	50.8
**Age (years)**				
16–29	57.4	28.2	30.6	24.3
30–39	19.5	20.8	25.8	19.7
40–49	12.6	20.1	21.0	19.0
50–59	6.7	14.5	13.8	15.6
60+	3.9	16.4	8.8	21.4
**Marital status**				
Married (registered)	26.4	43.3	42.0	56.7
Never married	63.8	35.7	41.3	29.2
Divorced/separated/widowed	9.7	20.9	16.4	13.9
**Highest qualification**				
University degree	14.3	16.7	16.2	24.8
TAFE certificate/diploma^d^	37.9	35.2	38.5	32.9
Higher School Certificate/Year 12	21.1	13.7	14.1	13.1
School Certificate/Year 10	16.8	18.0	17.7	16.6
Primary/Year 7–9/Other	9.6	15.8	12.7	11.6
**Household income**				
< $20,000	13.8	26.4	18.6	17.0
$20,000–$39,999	13.6	16.6	17.2	15.1
$40,000–$59,999	15.2	14.9	16.0	15.2
$60,000+	37.0	20.0	30.2	33.1
Don't know/refused	20.4	22.1	18.1	19.6
**Employment status**				
Employed	73.5	50.7	66.2	61.1
Unemployed or in unpaid work	26.2	48.8	33.4	38.9
**Area of residence**				
Urban area	64.4	70.0	68.5	69.8
Rural area	35.6	30.0	31.5	30.2

Source: New South Wales Population Health Survey, Centre for Epidemiology and Research, NSW Department of Health

^a ^Based on survey question: 'On a day when you drink alcohol, how many standard drinks do you usually have?' Usually drinking 5 or more (for females) and 7 or more (for males) were identified as high risk alcohol consumption.

^b ^Based on a Psychological Distress Scale (using the K10) of 22 or more.

^c ^Current smokers were categorized by respondents who indicated they smoked daily or smoked occasionally.

^d ^Technical and Further Education College certificate or diploma

### Prevalence of risk factors

High-risk alcohol consumption (usually drinking 5 or more standard drinks if female; 7 or more if male) was reported by nearly one-fifth (19.6%) of males and just under one-sixth (15.4%) of females aged 16–29 years, as seen in Table 2. This high risk drinking was reported approximately three times more frequently among respondents aged 16 to 29 years than those aged 30 years or more. Nearly twice as many females (33.0%) as males (18.7%) were non-drinkers.

**Table 2:** Alcohol consumption, smoking status, psychological distress and emergency department presentation by sex and age group in NSW, 2002–2004

**Males**	**16–29 years % (n = 2,258)**	**30–59 years % (n = 7,122)**	**60+ years % (n = 5,076)**	**Total Males % (n = 14,456)**
**Alcohol consumption**^a^				
High risk (7+ males)	19.6	6.3	2.2	8.8
Risky (5–6 males)	15.2	9.6	4.9	10.0
Low risk (1–4 males)	45.6	67.8	68.7	62.5
Non-drinker	19.5	16.3	24.1	18.7
**Psychological distress**				
High	8.8	8.3	6.0	7.9
Moderate	19.9	17.0	14.7	17.3
Low	71.3	74.7	79.3	74.8
**Smoking status**				
Current smoker	28.2	26.5	10.1	23.6
Ex-smoker	27.3	38.4	58.6	39.8
Never smoker	44.4	35.1	31.2	36.6
**ED presentation**	17.4	13.1	15.6	14.7

**Females**	**16–29 years % (n = 2,876)**	**30–59 years % (n = 9,973)**	**60+ years % (n = 7,669)**	**Total Females % (n = 20,518)**

**Alcohol consumption**^a^				
High risk (5+ females)	15.4	4.3	0.5	6.1
Risky (3–4 females)	22.4	15.1	4.5	14.4
Low risk (1–2 females)	31.6	51.5	50.2	46.4
Non-drinker	30.6	29.1	44.8	33.0
**Psychological distress**				
High	12.7	10.6	8.0	10.5
Moderate	23.4	18.5	18.0	19.6
Low	63.9	70.8	74.0	69.9
**Smoking status**				
Current smoker	25.8	21.3	7.5	19.3
Ex-smoker	27.1	33.8	32.9	32.0
Never smoker	47.0	44.9	59.5	48.7
**ED presentation**	15.7	12.2	13.9	13.4

**Persons**	**16–29 years % (n = 5,134)**	**30–59 years % (n = 17,095)**	**60+ years % (n= 12,745)**	**Total Persons % (n = 34,974)**

**Alcohol consumption**^a^				
High risk (5+ females)	17.5	5.3	1.3	7.4
Risky (3–4 females)	18.8	12.4	46.8	12.3
Low risk (1–2 females)	38.6	59.6	58.8	26.0
Non-drinker	25.1	22.7	35.2	54.3
**Psychological distress**				
High	10.7	9.5	7.1	9.3
Moderate	21.7	17.8	16.5	18.5
Low	67.6	72.8	76.4	72.3
**Smoking status**				
Current smoker	27.0	23.9	8.7	21.4
Ex-smoker	27.2	36.1	44.8	35.8
Never smoker	45.7	40.0	46.4	42.8
**ED presentation**	16.6	12.6	14.7	14.0

Source: New South Wales Population Health Survey, Centre for Epidemiology and Research, NSW Department of Health.

^a ^Based on survey question: 'On a day when you drink alcohol, how many standard drinks do you usually have?'

High psychological distress was more often reported among females (10.5%) than among males (7.9%) with women aged 16 to 29 years reporting the highest rate of psychological distress (12.7%). Current smoking was reported most commonly among people aged 16 to 29 years, with males (28.2%) reporting higher rates than females (25.8%) in this age group. For both males and females, high-risk alcohol consumption, high psychological distress and being a current smoker decreased with age. Young males aged 16 to 29 years were the most likely (17.4%) to have presented to an emergency department in the last 12 months across all age and sex groups.

### Alcohol consumption and emergency department presentations

Persons who engage in high risk alcohol consumption or who are current non-drinkers are more likely than low risk drinkers to have presented to an emergency department in the past 12 months. The prevalence rate ratio for high risk alcohol consumption and emergency department presentations, after controlling for age, sex, psychological distress, smoking status, household income and marital status is 1.36 (95% CI: 1.12–1.65) compared to lighter drinkers, while for current non-drinkers it is 1.25 (95% CI: 1.16–1.35).

Table 3 demonstrates that when separated by sex and age groups, these findings were not significant for males but remained significant across all age groups for females. In particular, women aged 60 years or more who reported they usually drank at high risk levels were more than three times (PRR: 3.24, 95% CI: 1.38–7.61) as likely to have presented to an emergency department in the past 12 months than women of the same age group who were low risk drinkers.

**Table 3:** Cox's proportional hazard regression model for alcohol consumption and emergency department presentation by sex and age group in NSW, 2002–2004

Sex, age and alcohol consumption^a^	Emergency Department Presentation Prevalence Rate Ratio^b ^(95% CI)			
**Males**	**16–29 years**	**30–59 years**	**60+ years**	**Total Males**

High risk	1.27	0.93	0.63	1.2
(7+ drinks males)	(0.76, 2.11)	(0.62, 1.40)	(0.25, 1.62)	(0.94, 1.54)
Risky	0.93	0.85	0.87	0.9
(5–6 drinks, males)	(0.53, 1.61)	(0.57, 1.27)	(0.51, 1.43)	(0.64, 1.26)
Low risk	1	1	1	1
(1–4 drinks, males)	0.83	1.28	1.34	1.18
	(0.55, 1.24)	(0.91, 1.80)	(0.93, 1.93)	(0.97, 1.45)

**Females**	**16–29 years**	**30–59 years**	**60+ years**	**Total Females**

High risk	1.44*	1.49†	3.24*	1.61†
(5+ drinks, females)	(1.03, 1.99)	(1.19, 1.86)	(1.38, 7.61)	(1.30, 2.01)
Risky	0.85	1.21	1.14	1.09
(3–4 drinks, females)	(0.59, 1.23)	(0.95, 1.55)	(0.58, 2.22)	(0.89, 1.32)
Low risk	1	1	1	1
(1–2 drinks, females)				
Non-drinker	1.36*	1.35†	1.42†	1.39†
	(1.06, 1.74)	(1.22, 1.49)	(1.18, 1.70)	(1.30, 1.49)

**Persons**	**16–29 years**	**30–59 years**	**60+ years**	**Total Persons**

High risk	1.32	1.14	1.1	1.36†
(5+ female, 7+ male)	0.89, 1.96	0.94, 1.38	0.78, 1.55	1.12, 1.65
Risky	0.83	1.01	0.97	0.96
(3–4 female, 5–6 male)	0.60, 1.15	0.90, 1.15	0.66, 1.44	0.86, 1.08
Low risk	1	1	1	1
(1–2 female, 1–4 male)				
Non-drinker	1.07	1.27†	1.33†	1.25†
	0.86, 1.34	1.08, 1.49	1.13, 1.55	1.16, 1.35

Source: New South Wales Population Health Survey, Centre for Epidemiology and Research, NSW Department of Health.

^a ^Based on survey question: 'On a day when you drink alcohol, how many standard drinks do you usually have?'

^b ^Controlling for psychological distress, smoking status, household income and marital status.

^†^P < 0.01. * P < 0.05.

### Psychological distress and emergency department presentations

The more psychologically distressed a person, the more likely he or she is to have presented to an emergency department. The prevalence rate ratio, after adjusting for age, sex, alcohol consumption, smoking status, household income and marital status, indicated that those who have a high level of psychological distress are nearly twice as likely to have presented to an emergency department (PRR = 1.84, 95% CI: 1.65–2.06) while those experiencing moderate psychological distress are also more likely to have presented to an emergency department (PRR = 1.35, 95% CI: 1.20–1.51) than those with low psychological distress, as seen in Table 4.

**Table 4:** Cox's proportional hazard regression model for psychological distress and emergency department presentation by sex and age group in NSW, 2002–2004

**Sex, age and psychological distress**^a^	**Emergency Department Presentation Prevalence Rate Ratio**^b^**(95% CI)**			
**Males**	**16–29 years**	**30–59 years**	**60+ years**	**Total Males**

High	1.67* (1.07, 2.59)	2.46† (2.08, 2.91)	1.35 (0.77, 2.37)	1.96† (1.69, 2.28)
Moderate	1.32 (0.90, 1.94)	1.50† (1.31, 1.73)	1.26* (1.03, 1.54)	1.39† (1.20, 1.62)
Low psych distress	1	1	1	1

**Females**	**16–29 years**	**30–59 years**	**60+ years**	**Total Females**

High	1.35 (0.83, 2.20)	2.01† (1.68, 2.41)	1.87† (1.52, 2.30)	1.77† (1.54, 2.03)
Moderate	1.08 (0.74, 1.59)	1.47* (1.06, 2.03)	1.37* (1.03, 1.83)	1.32† (1.10, 1.57)
Low psych distress	1	1	1	1

**Persons**	**16–29 years**	**30–59 years**	**60+ years**	**Total Persons**

High	1.47* (1.12, 1.93)	2.19† (1.90, 2.51)	1.62† (1.29, 2.02)	1.84† (1.65, 2.06)
Moderate	1.19 (0.92, 1.53)	1.48† (1.28, 1.71)	1.30† (1.09, 1.55)	1.35† (1.20, 1.51)
Low psych distress	1	1	1	1

Source: New South Wales Population Health Survey, Centre for Epidemiology and Research, NSW Department of Health.

^a ^Psychological Distress Scale: Low = <16; Moderate = 16–21; High: 22+.

^b ^Controlling for alcohol consumption, smoking status, household income and marital status.

† P < 0.01. * P < 0.05.

The highest rates of psychological distress were reported in persons aged 30 to 59 years who were more than twice as likely to have presented to an emergency department as persons in the same age group with low distress scores. This finding was stronger for males (PRR = 2.46, 95% CI: 2.08–2.91) aged 30 to 59 years than for females (PRR = 2.01, 95% CI: 1.68–2.41).

### Smoking status and emergency department presentations

Being a current or an ex-smoker were also predictors of having presented to an emergency department in the last year. Table 5 demonstrates that current smokers were 1.4 times more likely (PRR = 1.38, 95% CI: 1.25–1.52) and ex-smokers were 1.3 times more likely (PRR = 1.30, 95% CI: 1.11–1.51) to report an emergency department presentation than non-smokers, after adjusting for age, sex, alcohol consumption, psychological distress, household income and marital status.

**Table 5:** Cox's proportional hazard regression model for smoking status and emergency department presentation by sex and age group in NSW, 2002–2004

**Sex, age and smoking status**	**Emergency Department Presentation Prevalence Rate Ratio**^a^**(95% CI)**			
**Males**	**16–29 years**	**30–59 years**	**60+ years**	**Total Males**

Current smoker	1.86* (1.14, 3.03)	1.28 (0.98, 1.68)	0.79 (0.61, 1.04)	1.34† (1.12, 1.61)
Ex-smoker	1.62† (1.21, 2.16)	1.21 (0.72, 2.03)	1.06 (0.83, 1.35)	1.28* (1.00, 1.64)
Never smoker	1	1	1	1

**Females**	**16–29 years**	**30–59 years**	**60+ years**	**Total Females**

Current smoker	1.78* (1.20, 2.65)	1.27 (1.00, 1.61)	1.14* (1.03, 1.25)	1.38† (1.32,,1.45)
Ex-smoker	1.45* (1.08, 1.95)	1.30* (1.04, 1.62)	1.17 (0.98, 1.41)	1.29† (1.10, 1.51)
Never smoker	1	1	1	1

**Persons**	**16–29 years**	**30–59 years**	**60+ years**	**Total Persons**

Current smoker	1.82† (1.32, 2.50)	1.29† (1.11, 1.50)	1.00 (0.90, 1.11)	1.38† (1.25, 1.52)
Ex-smoker	1.53† (1.22, 1.93)	1.26 (1.00, 1.60)	1.17† (1.08, 1.28)	1.30† (1.11, 1.51)
Never smoker	1	1	1	1

Source: New South Wales Population Health Survey, Centre for Epidemiology and Research, NSW Department of Health.

^a ^Controlling for alcohol consumption, psychological distress, household income and marital status.

† P < 0.01. * P < 0.05.

Among current smokers, the highest rates of emergency department presentations were found for persons aged 16 to 29 years, even after controlling for alcohol consumption and psychological distress.

Ex-smokers in this 16 to 29 age group also have a much higher rate of emergency department presentation than non-smokers or ex-smokers aged 30 years or more.

### Multiple risk factors and emergency department presentations

Table 6 shows that women aged 30 to 59 years who have all three risk factors (high risk alcohol consumption, high levels of psychological distress and being a current smoker) are more than three times (PRR = 3.44, 95% CI: 1.84–6.43) as likely to present to an emergency department than women of this age group who have none of these risk factors. This trend of increased emergency department presentations is generally higher for females than for males who report all three risk factors.

**Table 6:** Cox's proportional hazard regression model for multiple risk factors (alcohol consumption, high psychological distress, and current smoking) and emergency department presentations in NSW, 2002–2004

	**Emergency Department Presentation Prevalence Rate Ratio**^a^**(95% CI)**			
**Males**	**16–29 years**	**30–59 years**	**60+ years**	**Total Males**

High risk alcohol/current smoker/high psych distress	2.62† (1.45, 4.73)	1.69 (0.98, 2.94)	0.09 (0.01, 1.52)	1.95† (1.40, 2.72)
High risk alcohol/high psych distress	1.94* (1.01, 3.75)	1.10 (0.68, 1.78)	0.82 (0.28, 2.38)	1.48* (1.02, 2.15)
High risk alcohol/current smoker	3.20 (0.67, 15.16)	1.60 (0.40, 6.41)	0 (0.00, 0.01)	2.25 (0.52, 9.71)
High psych distress/current smoker	3.28* (1.09, 9.85)	2.95† (2.11, 4.10)	0.54 (0.11, 2.67)	2.65† (1.98, 3.56)
None of these	1	1	1	1

**Females**	**16–29 years**	**30–59 years**	**60+ years**	**Total Females**

High risk alcohol/current smoker/high psych distress	2.19 (0.59, 8.15)	3.44† (1.84, 6.43)	1.00 (1.00, 1.00)	2.67* (1.35, 5.30)
High risk alcohol/high psych distress	2.26† (1.56, 3.28)	1.31* (1.05, 1.64)	0.99 (0.07, 13.48)	1.78† (1.34, 2.37)
High risk alcohol/current smoker	3.37* (1.27, 8.93)	2.59 (0.78, 8.64)	10.77† (4.42, 26.22)	3.21† (1.82, 5.67)
High psych distress/current smoker	1.66 (0.84, 3.25)	1.79* (1.19, 2.69)	2.58 (0.92, 7.26)	1.82† (1.50, 2.22)
None of these	1	1	1	1

**Persons**	**16–29 years**	**30–59 years**	**60+ years**	**Total Persons**

High risk alcohol/current smoker/high psych distress	2.38* (1.19, 4.76)	2.33† (1.54, 3.55)	0.10 (0.01, 1.70)	2.28† (1.57, 3.33)
High risk alcohol/high psych distress	2.09† (1.36, 3.21)	1.20 (0.90, 1.59)	0.94 (0.34, 2.58)	2.68† (1.35, 5.30)
High risk alcohol/current smoker	3.13† (1.45, 6.76)	2.07 (0.97, 4.42)	2.57 (0.43, 15.31)	1.63* (1.26, 2.10)
High psych distress/current smoker	2.32* (1.29, 4.17)	2.26† (1.65, 3.11)	1.60 (0.69, 3.73)	2.17† (2.00, 2.36)
None of these	1	1	1	1

Source: New South Wales Population Health Survey, Centre for Epidemiology and Research, NSW Department of Health.

^a ^Controlling for household income and marital status.

† P < 0.01. * P < 0.05.

Women aged over 60 years who reported high risk alcohol consumption and high psychological distress were more than ten times (PRR = 10.77, 95% CI: 4.42–26.22) the number of emergency department presentations than women of this age group without these risk factors. For persons aged 16 to 29 years, having high-risk alcohol consumption and being a current smoker doubles the risk of presenting to an emergency department. This risk is higher for young women aged 16 to 29 years (PRR = 2.26, 95% CI: 1.56–3.28) than for young men aged 16 to 29 years (PRR = 1.94, 95% CI: 1.01–3.75) though both are statistically significant. By contrast, young males aged 16 to 29 years who have both high psychological distress and are current smokers are at the highest risk (PRR = 3.28, 95% CI: 1.09–9.85) of presenting to an emergency department.

## Discussion

The present study found that high-risk alcohol consumption, high psychological distress and current smoking were all significantly and independently associated with a greater likelihood of presenting to an emergency department in the last year, after adjusting for age, sex, household income and marital status. When these three risk factors are combined, the rate of emergency department presentation is higher than for each risk factor on its own. Many of these findings were significantly different between sex and age groups. In particular, it was notable that young people aged 16–29 years had the highest rates of emergency department presentations and also the highest rates of high-risk alcohol consumption, having high psychological distress, and being a current smoker.

The results of this study are compatible with the theory of a u-shaped relationship between current alcohol consumption level and presentation at emergency departments; that is, current non-drinkers and high-risk drinkers have a higher risk of presenting than low risk drinkers. This u-shaped curve was only found to be significant for females, with the surprising finding that women over 60 years who usually drink alcohol at high risk levels are more than 3 times as likely to present to the emergency department than women of the same age who drink at low risk levels. When looking at women over 60 years who also have high psychological distress along with high risk drinking, the risk of presenting to an emergency department increases ten-fold. High-risk drinking among older people, particularly older women, appears to be an understudied issue. For this population, detection of alcohol-related problems is poor and brief interventions are few [22,23]. Although older people have generally lower rates of alcohol consumption, this study has identified the need for more research into the prevalence and effect of high-risk alcohol consumption on this population.

Frequent emergency department presenters have previously been found to be more likely to have high rates of psychological distress [24,25]. The strongest association in this study for high psychological distress and emergency department presentations was found for males aged 30–59 years, who were nearly two-and-a-half times more likely to have presented to an emergency department than males of the same age with low psychological distress scores. It is not clear from this research if participants were distressed due to mental health problems, and therefore presented to an emergency department more often, or whether they were distressed as a result of another health condition that caused them to present to the emergency department. Further research is needed to understand the reasons for this and to determine the need for and access to mental health services among this population.

This research also identified a high rate of emergency department presentations among current smokers aged 16 to 29 years. The emergency department offers the opportunity for accessing this young, risk-taking population by providing opportunistic screening and brief interventions. Such interventions could include smoking cessation, and should be designed to be quickly and effectively delivered in an emergency department setting [26-28].

This findings of this research identifies the need for services which integrate smoking, alcohol and mental health interventions in the emergency department setting. In particular, this research has helped to highlight the population groups who have the highest prevalence and combination of these risk factors and who are at risk of being more frequent users of emergency care. This can assist with the development of brief interventions in the emergency department setting which consider all of these areas of public health concern.

These findings also identify possible additional questions the New South Wales Population Health Survey might collect, such as questions about lifetime alcohol consumption, number of emergency department presentations in the last 12 months, and the reason for each presentation. With these additional questions, a more comprehensive investigation into the associations and interactions between high-risk alcohol consumption, high psychological distress, and current smoking with emergency department presentations could be undertaken. This future research should assist in assessing the potential role of the emergency department as a site for intervention for health risk behaviors.

There are a number of limitations to this study. First, it was an opportunistic study utilizing a data source designed and collected for a broader monitoring of population health. As a result, the nature and direction of the relationship between high-risk alcohol consumption, high psychological distress and current smoking with emergency department presentations cannot be determined. Respondents may have presented at an emergency department for a variety of reasons, and consumed more alcohol or tobacco or been distressed as a result of other health problems. Second, all survey data is self-reported, which may result in underestimations of emergency department presentation and of alcohol or tobacco consumption [29]. Third, it is not possible to distinguish between ex-drinkers and lifetime non-drinkers among survey respondents. Ex-drinkers may have stopped drinking alcohol due to health problems associated with their drinking and be more likely to need health services [8]. Fourth, the survey did not ask respondents about how frequently they presented to an emergency department or the reason for their presentation, so this could not be examined. Fifth, the results include the category of 'current smoker' which is acknowledged to include a broad spectrum of smoking levels. Further quantification of risk of attending the emergency department by the amount smoked was not evaluated.

## Conclusion

The combination of being a high-risk consumer of alcohol, having high psychological distress, and being a current smoker are associated with increased presentations to emergency departments, independent of age and sex. Further research is needed to enhance recognition of and intervention for these symptoms in an emergency department setting in order to improve patient health and reduce future re-presentations to emergency departments.

## Competing interests

The author(s) declare that they have no competing interests.

## Authors' contributions

DI conceived of the study, performed the statistical analysis and drafted the manuscript. ME participated in the design of the study, provided methodological and statistical analysis advice and commented on drafts of the manuscript. JC participated in the design of the study and in the writing of the manuscript. KMC participated in the design of the study and in the writing of the manuscript. All authors read and approved the final manuscript.

## Pre-publication history

The pre-publication history for this paper can be accessed here:


